# Complete chloroplast genome sequence and phylogenetic analysis of *Erysimum cheiranthoides*

**DOI:** 10.1080/23802359.2019.1660924

**Published:** 2019-09-06

**Authors:** Qingde Zhang, Yingxue Du, Shangyuan Bai, Wangjun Yuan

**Affiliations:** a Key Laboratory of Plant Stress Biology, School of Life Sciences, Henan University, Kaifeng, China;; b Institute of Pharmacy, Henan University, Kaifeng, China

**Keywords:** *Erysimum cheiranthoides*, chloroplast genome, phylogenomics

## Abstract

*Erysimum cheiranthoides* is commonly known as treacle-mustard or wormseed wallflower with value for reducing high temperature and inducing diuresis. Here, we characterized the complete chloroplast (cp) genome of *E. cheiranthoides* using genome skimming sequencing. The circular complete cp genome is 154,611 bp in length, containing a large single-copy (LSC) region of 83,809 bp, two copies of IR (26,475 bp each) regions, and a small single-copy (SSC) region of 17,852 bp. It comprises 113 unique genes, including 79 different protein-coding genes, 30 tRNA genes, and 4 rRNA genes with 19 duplicated genes in the IR regions. Phylogenetic analysis suggests that *E. cheiranthoides* is sister to *Olimarabidopsis pumila* with full support value in Brassicaceae.

*Erysimum cheiranthoides* L., commonly known as treacle-mustard or wormseed wallflower, is a species of *Erysimum* native to most of central and northern Europe and northern and central Asia (Blamey and Grey-Wilson 1989). Like other *Erysimum* species, *E. cheiranthoides* accumulates two major classes of defensive chemicals, glucosinolates, and cardiac glycosides, which make it become a Chinese crude drug used for reducing high temperature and inducing diuresis (Jiangsu New Medical College 1977). However, to date, there is still no complete cp genome characterized for *E. cheiranthoides*, even for the genus *Erysimum*. In the present study, we characterized the complete cp genome sequence of *E. cheiranthoides* (GeneBank accession number: MN207123) based on genome skimming data.

Total genomic DNA was isolated from silica-dried leaves of one wild *E. cheiranthoides* plant collected from Kaifeng (China; 114°31′44.89″E, 34°26′39.41″N) using modified CTAB reagent (Plant DNAzol, Shanghai, China) according to the manufacturer’s protocol. The voucher specimen was deposited at the herbarium of Henan University (accession number: 410222190406011LY). Illumina paired-end (PE) DNA library was prepared and sequenced in one lane of an Illumina Hiseq X10 by Beijing Genomics Institute (BGI, China) and obtained reads with length of 150 bp. Raw reads were trimmed using CLC genome workbench (CLC Inc., Rarhus, Denmark), the remaining reads were de novo assembled using GetOrganelle pipeline with SPAdes 3.10.1 as assembler (Jin et al. 2018). The software Geneious R11 (Biomatters, Auckland, New Zealand) was used to annotate the chloroplast genome of *E. cheiranthoides* following the description in Liu et al. (2017) and the circular plastome map was drawn utilizing the OrganellarGenomeDRAW tool (OGDRAW). Phylogeny relationships of Brassicaceae were inferred using the whole plastome sequences and maximum likelihood (ML) was implemented in RAxML-HPC v8.1.11 on the CIPRES cluster (Miller et al. 2010).

The complete chloroplast genome of *Erysimum cheiranthoides* is 154,611 bp in length and shares the common feature of comprising two copies of IR (26,475 bp each) that divide the genome into two single-copy regions (LSC 83,809 bp; SSC 17,852 bp). The overall GC content of the total length, LSC, SSC, and IR regions is 36.5, 34.4, 29.5, and 42.4%, respectively. Within the chloroplast genome of *E. cheiranthoides,* there are 113 genes, including 79 protein-coding genes, 30 tRNA genes, 4 rRNA genes, and 19 duplicated genes. Among the 113 unique genes, six tRNA and nine protein-coding genes contain one intron and three (*rps*12, *clp*P, and *ycf*3) contain two introns. The constructed phylogeny indicated that 46 representative species from Brassicaceae formed a highly supported clade and *E. cheiranthoides* is sister to *O. pumila* with full support value (Figure 1).

**Figure 1.**
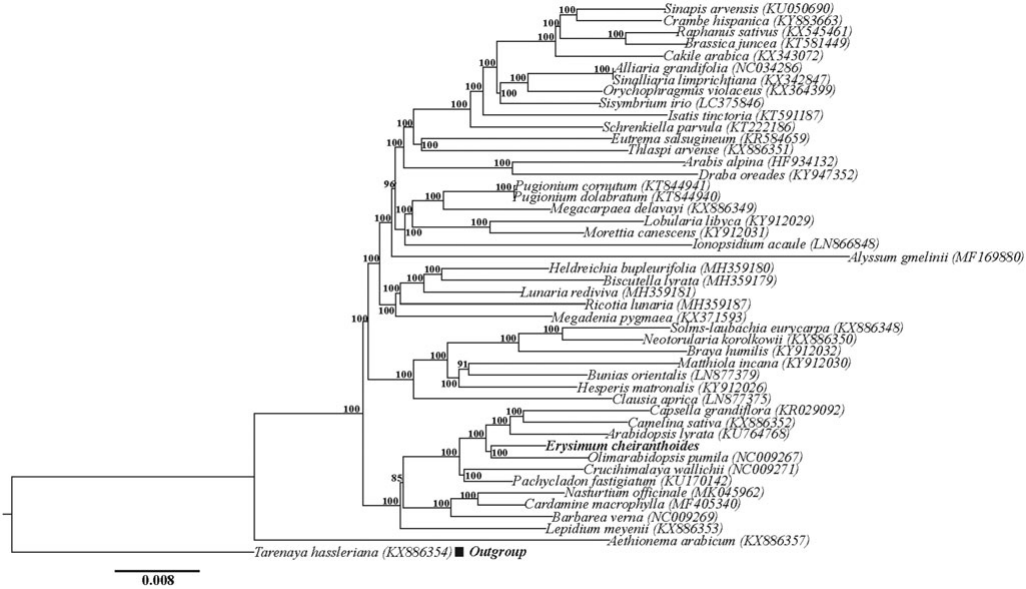
Phylogenetic relationships of Brassicaceae inferred based on whole cp genome sequences. Numbers above the branches represent bootstrap values from maximum likelihood analyses. GenBank accession numbers of taxa are shown after the species name.

In conclusion, the complete chloroplast genome of *E. cheiranthoides* is reported for the first time in this study. It will pave the way for future research to understand the genomic information of the chloroplasts of the genus *Erysimum* and this chloroplast resource could also be utilized on the phylogeny, DNA barcoding, and conservation genetics.
